# Effect of Reheating Temperature on the Microstructure and Properties of Cu-Containing 440 MPa Grade Non-Tempered Ship Plate Steel

**DOI:** 10.3390/ma17071630

**Published:** 2024-04-02

**Authors:** Dian Zhang, Feng Chai, Xiaobing Luo, Zhongran Shi

**Affiliations:** Central Iron & Steel Research Institute, Haidian District, Beijing 100082, China

**Keywords:** ship plate steel, reheating temperature, original austenite, precipitated phase, precipitation strength

## Abstract

This study investigated the effects of reheating temperature on the microstructure and mechanical properties of Cu-containing 440 MPa grade non-tempered ship plate steel. The mechanical properties test, thermodynamic simulation, optical microscopy, scanning electron microscopy, transmission electron microscopy, and other tests were performed. The results revealed that with increasing reheating temperature, the ferrite grain size of Cu-containing 440 MPa non-tempered ship plate steel increased. Also, with increasing reheating temperature, the size of copper particles and niobium–titanium composite precipitates in the original austenite decreased. Consequently, this led to a weakening of the pinning effect on the original austenite and an increase in the size of the transformed ferrite grains. Moreover, with increasing reheating temperature, the number of Cu precipitates in the test steel after air cooling and rolling increased, while the size of the precipitates decreased, thereby weakening the solid solution strengthening effect of Cu, and precipitation was enhanced. Additionally, as the reheating temperature increased, the tensile strength and yield strength of the air-cooled test steel after rolling increased, while the impact toughness decreased.

## 1. Introduction

Ship plate steel is mainly used for constructing ship hulls, decks, and other components. Moreover, ship plate steel is used in complex marine environments that require specific criteria regarding structural function, composition design, and mechanical properties. Therefore, ship plate steel must exhibit high strength and toughness, excellent weldability, and corrosion resistance to meet specific requirements [1,2,3]. The traditional 440 MPa grade ship plate steel mainly incorporates Ni, Cr, and Mo alloy elements and undergoes quenching and tempering processes to achieve tempered martensite [4]. However, the high-carbon equivalent in traditional ship plate steel poses challenges in welding, increases costs, and results in unstable performance. To address these issues, high-strength low-alloy (HSLA) series ship plate steel was developed. The HSLA series of Cu-containing ship plate steel incorporates ultra-low-carbon micro-alloying and undergoes controlled rolling and cooling processes. Moreover, this steel series is characterized by easy weldability, good strength, and toughness matching [5]. A representative example of this series is the 440 MPa grade HSLA65 steel. The process characteristics of Cu-containing ship plate steel involve the addition of copper to enhance the low strength of steel resulting from a low carbon content. Additionally, higher copper contents are introduced to induce precipitation strengthening and address the loss associated with low carbon. The combination of these enhancements with controlled rolling and cooling processes enabled the plate steel to meet the high strength and toughness requirements [3,6]. Copper-containing ship plate steel exhibits a significant precipitation strengthening effect capable of achieving carbon-independent strengthening. The formation of ultrafine Cu-rich precipitates not only enhances the strength, wear resistance, fatigue resistance, impact resistance, and weldability of the steel, but also contributes to improved corrosion resistance [7,8]. The copper in austenite exhibits very high solid solubility. With decreasing temperature, the solid solubility of copper sharply decreases, leading to significant copper precipitation. The copper-containing phase improves the lattice strength of the test steel in the ferrite matrix [9]. Northwest University developed a high-performance low-carbon weathering steel NUCu70 W using HSLA steel. The steel was designed to eliminate elements such as Cr and Mo and achieve a yield strength of at least 70 ksi (483 MPa) without quenching and without tempering under hot rolling and air cooling conditions [10]. NUCu70 W steel exhibited high strength, excellent ductility, and fracture toughness owing to the presence of nanoscale coherent Cu-alloying precipitates and their interaction with spiral dislocations in the ferrite matrix [11,12]. Additionally, the NuCu series steel is designed to minimize the use of costly alloying elements and utilizes cost-effective processing technology to streamline production.

Currently, research on Cu-containing ship plate steel has mainly focused on examining the influence of aging time and temperature on Cu precipitation. Conversely, studies on the precipitation of Cu after air cooling and rolling with simple heat treatment are few. Alloying elements such as Ni, Ti, and Nb were modified using NuCu70 W steel, and the influence of the second-phase precipitates on the microstructure and mechanical properties of the test steel was investigated. In a non-quenched and tempered state, the new 440 MPa hull steel met the performance criteria of yield strength ≥440 MPa, tensile strength ≥550 MPa, and impact energy absorption at −40 °C ≥50 J. Compared with the traditional 440 MPa hull steel, the test steel is characterized by a simple production process and low cost.

In this study, the changes in the microstructure and properties of non-tempered ship plate steel at different reheating temperatures were investigated. Second-phase precipitation was examined through thermodynamic calculations and transmission. The effects of reheating temperature on grain sizes and precipitated phases were evaluated. Moreover, the impacts of grain sizes and precipitated phases on the microstructure and mechanical properties of the test steel were assessed. Furthermore, the suitable reheating temperature for the precipitation of microalloying elements was identified.

## 2. Test Materials and Methods

The chemical compositions (mass fraction, %) of the test steel comprised C 0.059, Si 0.46, Mn 0.52, P 0.005, S 0.001, Ti 0.017, Nb 0.056, Cu 1.29, Ni 1.06, Al 0.036, and N 0.001. The test steel underwent smelting in a 50 kg vacuum melting furnace. Subsequently, the resulting ingot was forged into a slab with dimensions of 7mm × 120 mm× 150 mm (thickness × width × length). The slab was then heated to 1150 °C to achieve a solid solution of alloying elements and held for 2 h to homogenize. The initial forging temperature was set to 1150 °C, and the final forging temperature was set above 950 °C. After the forging process, the steel underwent air cooling. Then, the test steel was reheated to 1000 °C, 1150 °C, and 1200 °C in the warm furnace (unloaded with the furnace material) and held for 30 min after burning. Upon discharge from the furnace, the test steel underwent rough rolling, followed by five passes of finishing rolling. After rolling, the steel exhibited a final thickness of 12 mm, and the last rolling temperature was recorded at 830 °C, followed by air cooling.

The tested steel was used to process thermal simulation samples with dimensions of Φ10 × 15 mm. These samples were heated to temperatures ranging from 1000 °C to 1250 °C using a thermal simulation testing machine (Gleeble 3800, Dynamic Systems Inc., Albany, NY, USA). The temperature gradient was increased to 50 °C. Six samples were maintained for 5 min and then quenched in ice water for cooling. Subsequently, these samples were cut at the location of the thermocouple for further analysis. The original austenite grain boundary was corroded using a solution of picric acid and detergent. The original austenite grain size was determined by visually counting using an optical microscope. Samples were extracted from the thermal simulation specimens, and a Ni mesh was used to retrieve the carbon film. The precipitated phase was observed via transmission electron microscopy (Tecnai TEM G2, FEI Company, Hillsboro, OR, USA).

A metallographic sample was extracted from the test steel plate, polished perpendicular to the rolling direction, corroded with 4% nitric acid alcohol, and analyzed through optical microscopy (Olympus BX41,Olympus Corporation Company, Hamburg, Germany). The microstructure of the experimental steel was examined through scanning electron microscopy (Quanta 650, FEI Company, Hillsboro, OR, USA). Samples designed for a Transmission Electron Microscope (TEM, FEI Company, Hillsboro, OR, USA) were extracted from the metallographic samples, and the plane of observation was set perpendicular to the rolling direction. The cut sheet was mechanically ground to a thickness of 50 μm, and ion-thinned perforations were performed using an electrolytic double-spray RL-1 device. Subsequently, TEM analysis was performed using a transmission electron microscope (Tecnia F20 and H800). Ultrafine laths were examined through field emission transmission electron microscopy (H-800, 200 KV) using an electrolytic double spray in a 10% perchloric acid solution at −20 °C. The dislocation density was measured with an X-ray diffractometer (Model D8 Advance, Bruker Company, Bremen, Germany) operated at 35 kV using a Co target. The sample was processed at a scanning rate of 2°/min.

## 3. Result and Discussion

### 3.1. Effect of Reheating Temperature on Mechanical Properties

**Figure 1:**
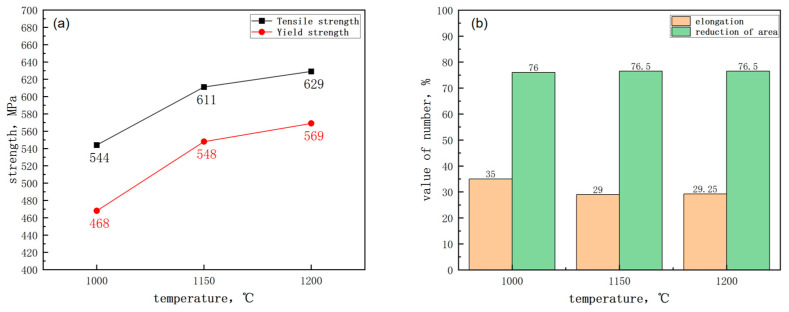
Effect of reheating temperature on strength of air-cooled test steel after rolling: (**a**) strength (**b**) elongation and reduction of area.

**Figure 2:**
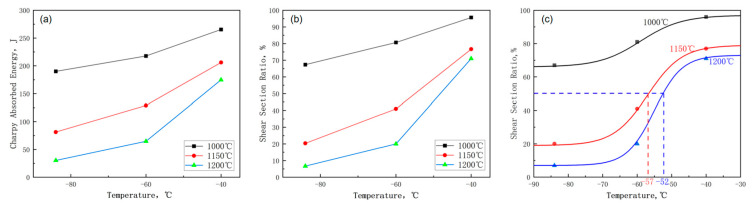
Effect of reheating temperature on toughness of air-cooled test steel after rolling: (**a**) impact energy (**b**) fiber ratio (**c**) ductile–brittle transition.

Figure 1 and Figure 2 show the variation in the mechanical properties of the test steel with temperature. The diagrams revealed that the yield strength of the test steel exceeded 440 MPa, and the impact energy at −40 °C exceeded 50 J, meeting the performance criteria for the new generation of 440 MPa hull steel. With increasing reheating temperature, both the tensile strength and yield strength of the test steel increased, while the elongation decreased (Figure 1). The change in reheating temperature slightly influenced the reduction of area in the specimen. With increasing reheating temperature, the impact energy and section fiber ratio of the test steel decreased (Figure 2). This indicates a decrease in the toughness of the test steel with increasing reheating temperature. The area fraction of the broken fiber was measured at different test temperatures, and FATT (fracture appearance transition temperature) 50, identified through Boltzmann function fitting, served as the ductile–brittle transition temperature. The ductile–brittle transition temperatures at 1150 °C and 1200 °C were −57 °C and −52.5 °C, respectively (Figure 2c). The ductile–brittle transition temperature at 1000 °C was significantly lower than that at 1150 °C and 1200 °C.

### 3.2. Microstructure

**Figure 3:**
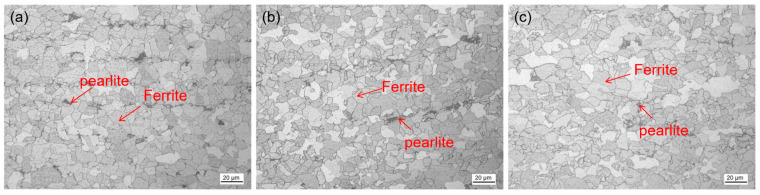
Metallographic microstructure of air-cooled test steel after rolling at different reheating temperatures: (**a**) 1000 °C; (**b**) 1150 °C; (**c**) 1200 °C.

**Figure 4:**
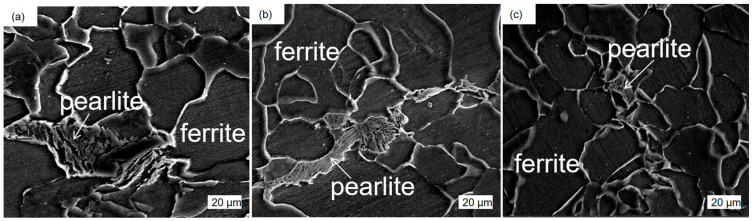
Scanning microstructure of air-cooled test steel after different reheating temperatures: (**a**) 1000 °C; (**b**) 1150 °C; (**c**) 1200 °C.

**Figure 5:**
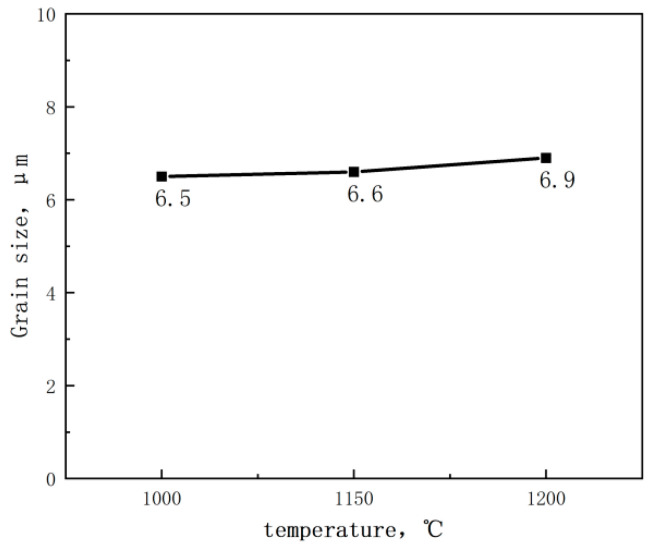
Change of grain size of test steel with reheating temperature.

The test steel underwent a rolling process at different reheating temperatures. The microstructure of the test steel mainly comprised ferrite with a small amount of pearlite, pearlitic content decreasing with increasing reheating temperature (Figure 3). The scanned structure of the test steel (Figure 4) at different reheating temperatures indicated that the test steel mainly consisted of ferrite and pearlite. The grain size of the metallographic structure was determined through a cross-section method. Figure 5 shows the variation in the grain size of the test steel with reheating temperature. The grain size increased with increasing temperature.

### 3.3. Effect of Different Reheating Temperatures on the Strength and Toughness of Test Steel

**Figure 6:**
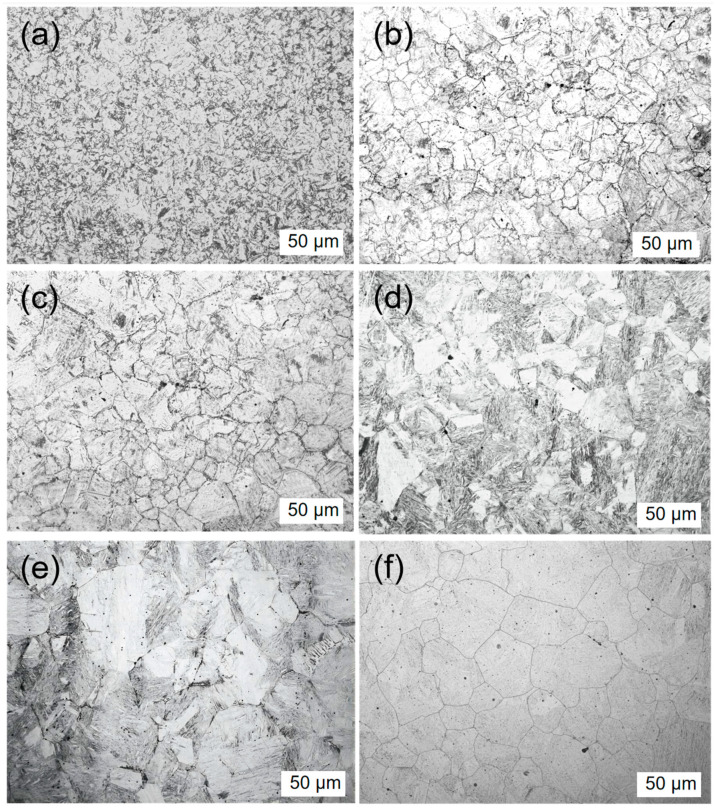
Primary austenite morphology of test steel with different reheating temperature: (**a**) 1000 °C, (**b**) 1050 °C, (**c**) 1100 °C, (**d**) 1150 °C, (**e**) 1200 °C, (**f**) 1250 °C.

**Figure 7:**
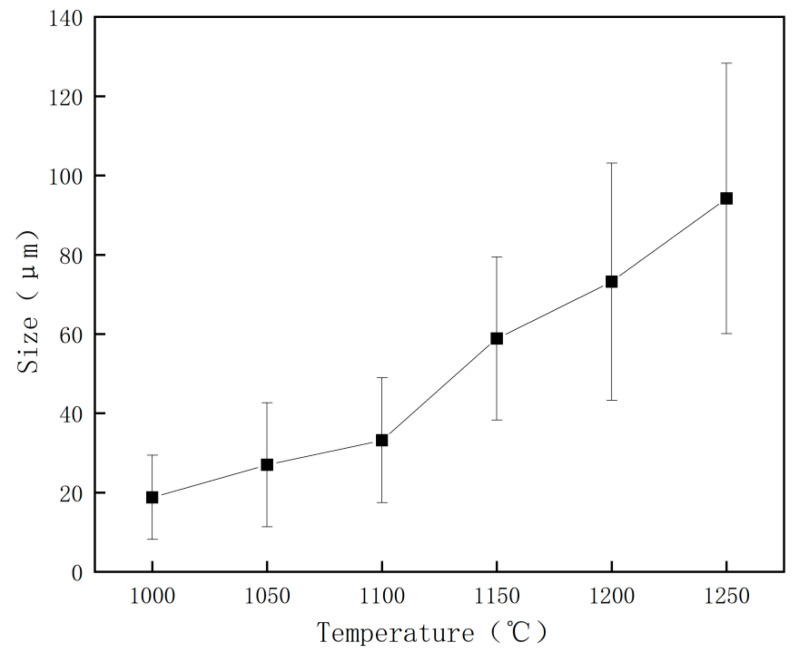
Effects of different reheating temperatures on the original austenite grain size of the test steel.

The grain size of the original austenite of the test steel played a vital role in determining the size of the ferrite matrix in the subsequent microstructure transformation, thereby influencing the toughness of the test steel. Figure 6 and Figure 7 show the changes in the original austenite microstructure and the average grain size with different reheating temperatures. As the reheating temperature increased, the average grain diameter of the original austenite increased, and the grains gradually assumed a flat and uniform shape. Additionally, the grain boundaries gradually transitioned from bending to a flat configuration. As the reheating temperature increased from 1100 °C to 1150 °C, the grain size of the original austenite significantly increased, leading to austenite grain coarsening at 1150 °C. 

In Figure 5, Figure 6 and Figure 7, both ferrite grain size and austenite grain size increase with the increase of reheating temperature. The larger the austenite grain size, the larger the size of the ferrite grains that form during the subsequent tissue transformation. With the increase of reheating temperature, the change of austenite grain size is obvious; the change of ferrite grain size is not very obvious, but there is still a tendency of growth; therefore, there is a correlation between ferrite and austenite grain sizes.

**Figure 8:**
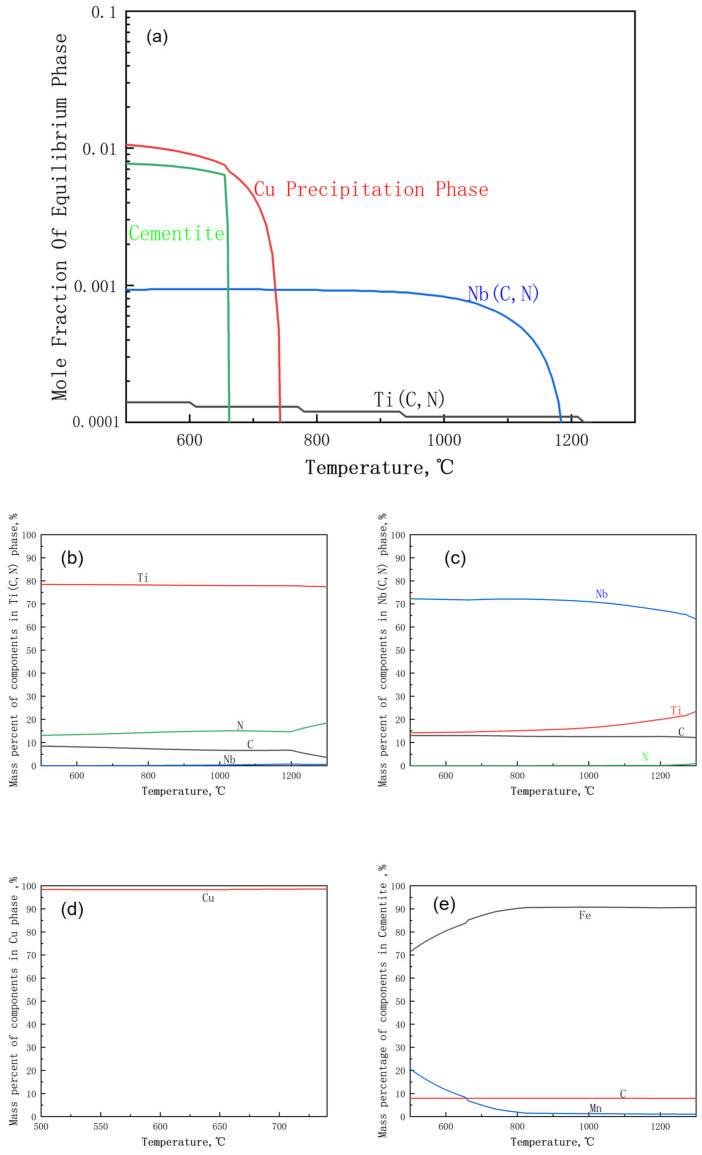
Changes in the molar fraction of the precipitated phase of the test steel with temperature and the mass percentage of each component in different precipitated phases: (**a**) changes in the molar fraction of the precipitated phase of the test steel with temperature; (**b**) the mass percentage of each component in Ti (C, N) precipitates; (**c**) the mass percentage of each component in the Nb (C, N) precipitates; (**d**) the mass percentage of each component in the Cu precipitated phase (**e**) the mass percentage of each component in the cementite precipitated phase.

The effect of reheating temperature on the precipitation behavior of the test steel was assessed using Thermo-Calc software 2022.1.93985-389. Figure 8 shows the changes in the molar fraction of the precipitated phase in the test steel with temperature. The second phase in the test steel mainly comprised the cementite phase, Cu precipitated phase, and (Ti, Nb) (C, N) precipitated phase. In the test steel, Ti and N exhibited strong affinity. The lower N content in the test steel compared with the ideal chemical ratio Ti/N (3.4) led to an incomplete precipitation of Ti. Therefore, Ti and Nb reacted with C to form TiC and NbC. The TiN, TiC, NbN, and NbC compounds all exhibited a face-centered cubic structure, facilitating their interaction and the formation of multiple carbon–nitrogen composite phases. At a temperature below 745 °C, a Cu precipitated phase was formed (Figure 8a). The mass percentage analysis of each precipitated phase (Figure 8) revealed that in the temperature range of 1000–1300 °C, the primary precipitated phase in the test steel was mainly titanium–niobium composite carbonitride (Ti, Nb) (C, N). Moreover, the precipitated phase in the original austenite of the test steel mainly comprised titanium–niobium composite carbonitride.

Grain growth is a continuous process involving the outward migration of grain boundaries. At its core, this process involved the movement of atoms through the diffusion interface, and the diffusion coefficient at the interface exhibited an exponential relationship with temperature. The grain growth process was mainly influenced by temperature [13,14,15]. The solid solubility formula for copper atoms in austenite is expressed as $lg\left(Cu\right)=2.652-\frac{2462}{T}$ [16]. According to the formula, the solid solubility of copper in austenite increases as the heating temperature increases, leading to a gradual decrease in the copper-containing phase. Large-scale dissolution of copper-containing phases into austenite reduces the hindrance to grain growth and promotes austenite grain growth [17,18].

**Figure 9:**
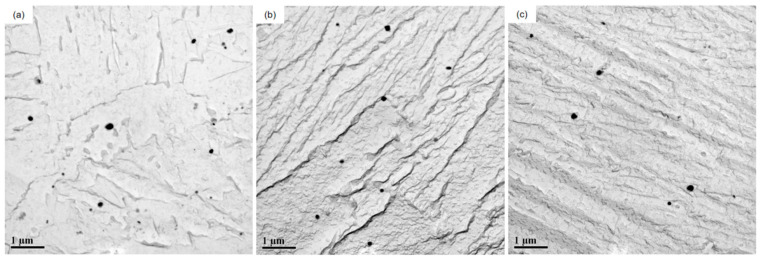
Experimental results of carbon film replica transmission of original austenite of experimental steel at different reheating temperatures: (**a**) 1000 °C; (**b**) 1150 °C; (**c**) 1200 °C.

**Figure 10:**
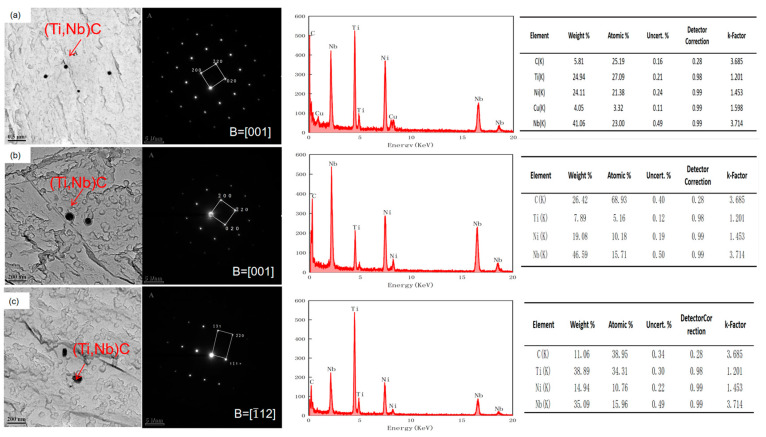
Precipitated particle morphology, diffraction spot and energy spectrum of original austenite of test steel at different reheating temperatures: (**a**) 1000 °C, (**b**) 1150 °C, (**c**) 1200 °C.

Figure 9 shows the distribution of the precipitated phase within the original austenite of the experimental steel at different reheating temperatures. The diagram provides a visual representation of the precipitated particles. The energy spectra of Figure 10a show that the precipitated phase in the austenite at a reheating temperature of 1000 °C is a carbide complex precipitate of titanium–niobium with undissolved Cu, and Figure 10b,c show that the precipitated phase in the austenite of the test steels at reheating temperatures of 1150 °C and 1200 °C is a carbide complex precipitate of titanium–niobium. The finely dispersed carbonitrides played a vital role in refining grains, enhancing precipitation strength, and controlling recrystallization [19,20,21,22,23]. A smaller size of the second-phase particles precipitated during the heating process indicated a greater number of these particles, which effectively pinned the austenite grain boundary to obtain relatively fine original austenite. The rolling process and the phase transformation contributed to grain refinement, resulting in a finer grain structure. As the reheating temperature increased, the composite carbide particles of titanium and niobium were gradually dissolved into the austenite grains, which weakened the hindrance to the growth of austenite grains, leading to the formation of coarse austenite grains. Moreover, the microstructural grains at room temperature exhibited a relatively large size. As the reheating temperature increased, the number of small-sized precipitated particles decreased, the size of precipitated particles increased, and their overall quantity decreased (Figure 9). Therefore, the use of a lower reheating temperature can effectively hinder the movement of original austenite grain boundaries caused by the second-phase particles, resulting in relatively fine original austenite. In the subsequent rolling process and phase transformation, the grains were further refined to obtain a finer grain structure, thereby improving the performance of the test steel.

**Figure 11:**
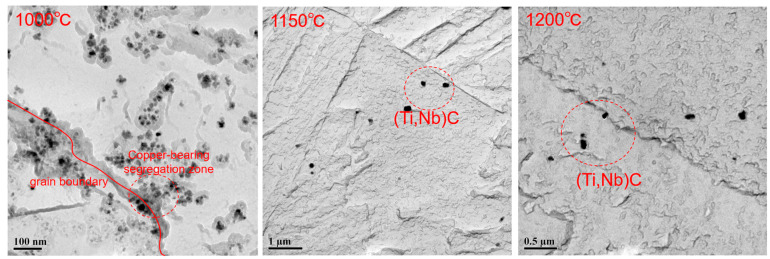
Morphology of precipitated particles in original austenite of test steel at different reheating temperatures.

At 1000 °C, Cu-rich zones were formed at grain boundaries (Figure 11). As the temperature increased, particularly during reheating at 1150 °C and 1200 °C, surpassing the melting point of Cu, the Cu in the original austenite gradually dissolved into the austenite matrix. This led to the elimination of the copper-rich zone at the grain boundary. 

According to the Orowan–Ashby precipitation strengthening mechanism [24], the increase in precipitation strengthening was mainly influenced by the size and volume fraction of the precipitated particles. A smaller size of precipitated particles and a higher volume fraction indicated a greater increase in precipitation strengthening. For a specific volume fraction, the higher dispersion of the precipitated particles resulted in a smaller size of the precipitated particles, leading to a more significant enhancement in precipitation strengthening.

**Figure 12:**
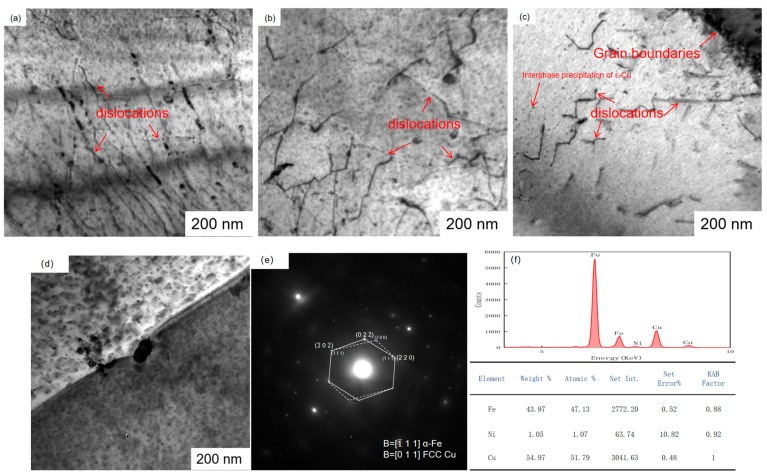
Transmission microstructure of air-cooled test steel after rolling at different reheating temperatures and the morphology, diffraction spot, and energy spectrum of the precipitated phase: (**a**) 1000 °C; (**b**) 1150 °C; (**c**) 1200 °C; (**d**) the morphology of the precipitated phase; (**e**) the diffraction spot of the precipitated phase; (**f**) the diffraction spot and energy spectrum of the precipitated phase.

Figure 12e shows the diffraction analysis of the precipitated phase. The energy spectrum and diffraction calibration revealed that the Cu precipitate was ε-Cu with a face-centered cubic structure (fcc). The diffracted electrons were incident along the crystal band axis [$\bar{1}$11] of the matrix α-Fe, and the reciprocal lattice (200) plane of the precipitated phase corresponded to the reciprocal lattice (02$\bar{2}$) plane of the matrix α-Fe. The ($\bar{1}$1$\bar{1}$) ($\bar{2}\bar{1}$1) planes of the precipitated phase were parallel to the (220) ($\bar{2}$0$\bar{2}$) planes of the matrix α-Fe, indicating a Kurdjumov–Sachs (K–S) orientation relationship between the precipitated phase Cu and the matrix α-Fe. The crystal plane spacing of the precipitated phase (200) was denoted as d (200) = 0.237 nm, and the calculated lattice constant of the precipitated phase was 0.353 nm, similar to the lattice constant of elemental Cu (0.3606).

Figure 12 shows a significant number of tangled dislocations near the grain boundary within the ferrite matrix. With increasing reheating temperature, the tangled dislocations in the test steel gradually decreased. X-ray diffraction experiments revealed dislocation densities of 7.1 × 10^8^, 5.3 × 10^8^, and 2.4 × 10^8^ cm^−2^ for different reheating experimental steels. The Cu precipitation on the ferrite matrix can be classified into several modes, such as precipitation on the dislocation line, supersaturated precipitation, and interphase precipitation. Some Cu-containing precipitates were observed on the dislocation sites within the steel, while others were present in the matrix of the test steel at 1150 °C and 1200 °C. With increasing reheating temperature, the dislocation density of the test steel decreased. Consequently, this led to fewer nucleation sites provided by dislocations, changes in the nucleation conditions of dislocations and interfaces, a reduction in the precipitation of dislocations, and an increase in the random and interphase distributions.

Goodman et al. [25] found that as the size of Cu precipitates exceeded 5 nm, the precipitates exhibited an incoherent fcc-type ε-Cu structure. As the size of Cu precipitates exceeded 10 nm, they exhibited a composition similar to that of pure Cu. The smaller precipitates featured a coherent body-centered cubic-Cu structure.

**Figure 13:**
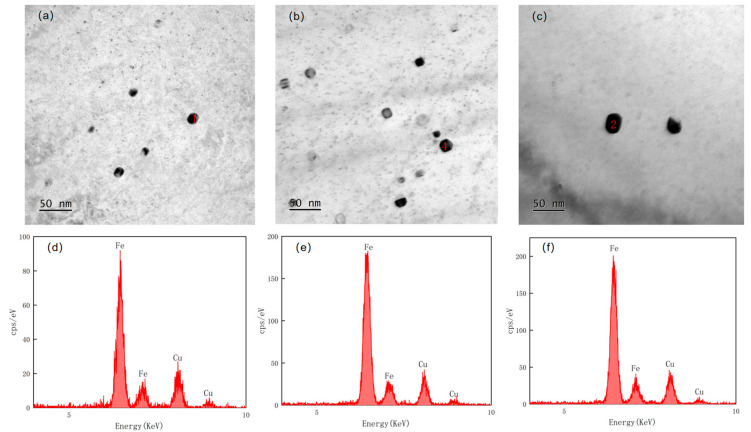
Precipitation phase morphology and corresponding EDS energy spectrum of air-cooled test steel after rolling at different reheating temperatures: (**a**,**d**) 1000 °C; (**b**,**e**) 1150 °C; (**c**,**f**) 1200 °C.

**Figure 14:**
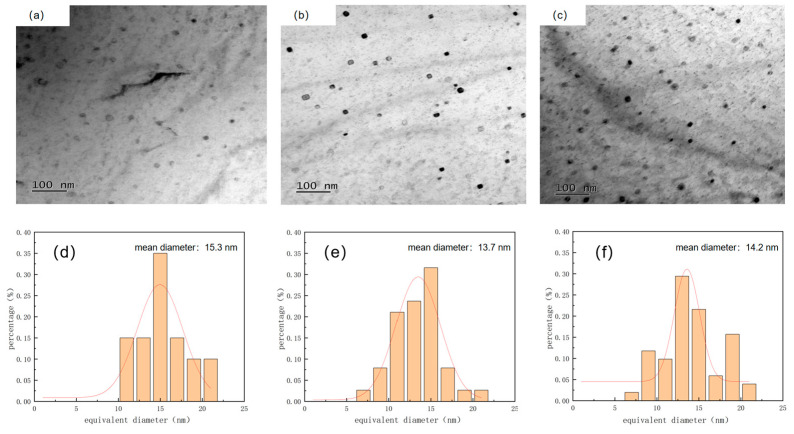
Number and size distribution of Cu-containing precipitates in air-cooled test steel after rolling at different reheating temperatures: (**a**,**d**) 1000 °C; (**b**,**e**) 1150 °C; (**c**,**f**) 1200 °C.

The precipitation distribution of Cu precipitates was mainly influenced by the nucleation mechanism and the migration mode of the γ/α interface. With increasing reheating temperature, the dislocation density of the transformed structure decreased, leading to changes in the precipitation conditions of Cu. Cu precipitation involved a nucleation-large phase transition [26]. The nucleation mechanisms of Cu include dislocation nucleation, interfacial nucleation, and homogeneous nucleation. The precipitated phase is easier to precipitate if its nucleation work and critical nucleation size are smaller. Dislocation and interface nucleation have much lower critical nucleation works than homogeneous nucleation, and this difference gets larger as the transformation temperature rises. The dislocation density of the test steel drops with rising reheating temperature, as do the nucleation sites that the dislocations give. Figure 13 and Figure 14 show variations in the distribution of Cu-containing precipitates at different reheating temperatures. At a reheating temperature of 1000 °C, the Cu-containing precipitates in the test steel exhibited a random distribution. At a reheating temperature of 1150 °C, the Cu-containing precipitates in the test steel featured a random and banded regular distribution. At a reheating temperature of 1200 °C, the distribution of Cu-containing precipitates in the test steel exhibited a predominantly banded regular and random distribution. The Cu precipitate exhibited an fcc structure (Figure 12). Cu-containing precipitates in the test steel exhibited variations in size and morphology at different reheating temperatures. The Cu-containing precipitates in the test steel reheated at 1000 °C exhibited a nearly circular shape, with an average size of 15.3 nm (Figure 13 and Figure 14). The copper-containing precipitates of the test steel reheated at 1150 °C featured a nearly round and oval shape, with an average size of 13.7 nm. The copper-containing precipitates of the test steel reheated at 1200 °C exhibited a nearly round and oval structure, with an average size of 14.2 nm. Notably, the predominant precipitated phases in the test steel were mainly Cu and niobium–titanium composite carbide precipitated phases. With increasing reheating temperature, the size of the Cu-containing precipitated phase gradually decreased, accompanied by an increase in the number and density of Cu-containing precipitated phases. This increase in the number of Cu-containing precipitates contributed to the precipitation strengthening and enhanced the strength of the test steel.

The free energy of precipitation for the ε-Cu phase in ferrite [27] can be described by ΔG=−59,214+54.99T, where T represents the thermodynamic temperature. At temperatures above 1077 K (804 °C), the free energy associated with the phase transformation of the ε-Cu precipitated phase (ΔGM) was positive, hindering the occurrence of ε-Cu precipitation. Conversely, temperatures below 1077 K promoted ε-Cu precipitation.

**Figure 15:**
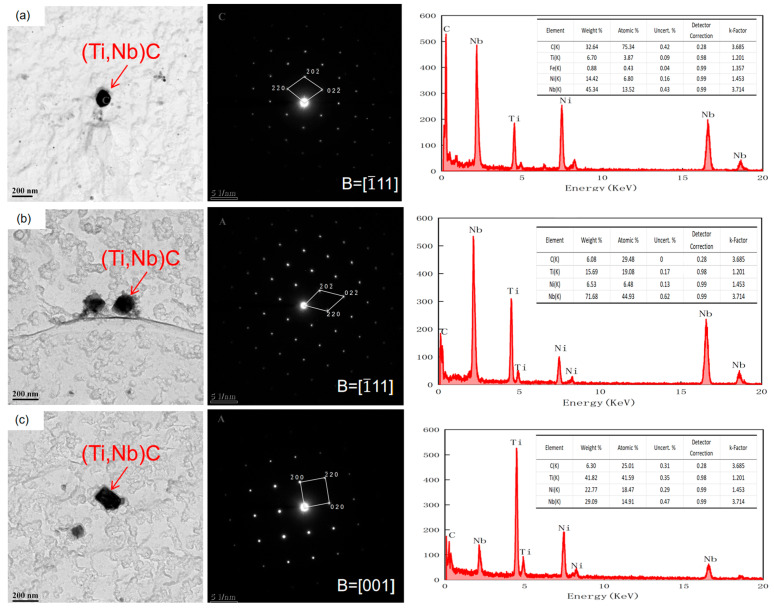
The morphology, diffraction spot, and energy spectrum of niobium–titanium carbide precipitates in air-cooled test steel after rolling at different reheating temperatures: (**a**) 1000 °C, (**b**) 1150 °C, (**c**) 1200 °C.

**Figure 16:**
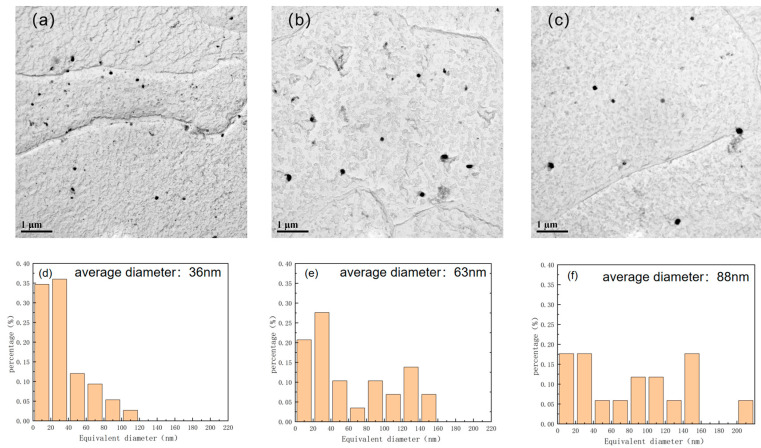
Number and size distribution of niobium–titanium carbide composite precipitates in air-cooled test steel after rolling at different reheating temperatures: (**a**,**d**) 1000 °C; (**b**,**e**) 1150 °C; (**c**,**f**) 1200 °C.

**Figure 17:**
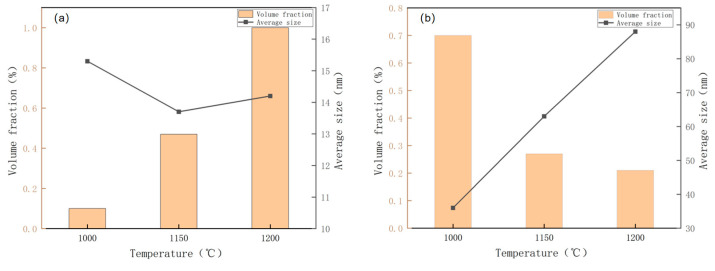
Size and volume fraction of precipitates in air-cooled test steel after rolling at different reheating temperatures: (**a**) Cu precipitates; (**b**) composite precipitates of Nb-Ti carbides.

The niobium–titanium carbide precipitates exhibited nearly circular, elliptical, and rectangular shapes (Figure 15 and Figure 16). As the reheating temperature increased, the proportion of rectangular precipitates increased. Additionally, with increasing reheating temperature, the average size of the precipitates increased. As the size of the precipitated particles exceeded 60 nm, the precipitation strengthening effect became considerably weak. Therefore, the primary factor influencing precipitation strengthening was mainly associated with the sizes of precipitated particles below 60 nm. The size of the precipitated phase in niobium–titanium composite carbides generally exceeded 60 nm. Therefore, the copper-containing precipitated phase emerged as the primary factor responsible for the change of precipitation strengthening. The size and volume fraction distributions of different precipitated phases are illustrated in Figure 17. In particular, the copper precipitated phase exhibited particle sizes below 20 nm. However, the size of the titanium–niobium carbide precipitated phase exceeded 30 nm, surpassing that of the copper precipitated phase.

The possible effect of reheating temperature on strength can be attributed to lattice resistance, fine grain strengthening, dislocation strengthening, solid solution strengthening, precipitation strengthening, and microstructure strengthening. The yield strength of ferritic steel was calculated using the Hall–Petch [28] formula, expressed as $\sigma_{y}=\sigma_{o}+△\sigma_{y}+△\sigma_{G}+△\sigma_{Dis}+△\sigma_{Orowan}$. Here, σ_y_ represents the yield strength, and σ_0_ denotes the matrix lattice resistance, also known as the P–N force. Typically, a low carbon steel featured a matrix lattice resistance P–N force of 48 MPa. ∆σ_y_, ∆σ_G_, ∆σ_Dis_, and ∆σ_Orowan_ represent the increases in yield strength due to solid solution strengthening, fine grain strengthening, dislocation strengthening, and precipitation strengthening, respectively.

The contribution of fine grain strengthening to yield strength can be determined using the formula $△\sigma_{G}=K_{y}d_{f}^{-1/2}$, where Ky is 17.4 MPa·mm^1/2^, and d_f_ represents the average grain size of ferrite. The experimental steel reheated at 1000 °C, 1150 °C, and 1200 °C exhibited average grain sizes of 6.5, 6.6, and 6.9 μm, respectively (Figure 5). Applying the provided formula, the experimental steel exhibited fine grain strengthening contributions of 216, 241, and 209 MPa.

The contribution of dislocation strengthening to yield strength can be determined using the formula $△\sigma_{Dis}=\alpha G\rho^{1/2}$. Here, α represents a crystallographic structure-dependent factor (α = 0.435), G denotes the shear modulus (8.3 × 104 MPa), b is the dislocation Burgers vector mode (0.248 × 10^−7^ cm), and ρ is the dislocation density, unit cm^−2^. X-ray diffraction experiments revealed dislocation densities of 7.1 × 10^8^, 5.3 × 10^8^, and 2.4 × 10^8^ cm^−2^ for reheating temperature of 1000 °C, 1150 °C, 1200 °C experimental steels. According to the provided formula, the dislocation strengthening contribution values for experimental steels at 1000 °C, 1150 °C, 1200 °C reheating temperatures were 24, 7, and 4 MPa.

The contribution of solid solution strengthening to yield strength can be calculated using the formula △σ_y_ = 37 [Mn] + 83 [Si] + 59 [Al] + 38 [Cu] + 3750 [N] + 4570 [C], where [M] denotes the mass fraction of solid solution elements, in %. Notably, the maximum equilibrium solubility of carbon in ferrite was 0.011%. Ti exhibited a strong affinity with N, but the N content in the test steel was significantly lower than the ideal chemical ratio Ti/N (3.42). Consequently, nearly all the N elements were present in the precipitates. The Cu-containing precipitated phases in the test steel at 1000 °C, 1150 °C, 1200 °C reheating temperatures featured volume fractions of 0.1%, 0.47%, and 1%. The solid solution Cu elements in the test steel at 1000 °C, 1150 °C, 1200 °C reheating temperatures exhibited mass percentages of 1.19%, 0.82%, and 0.29%. The chemical composition of the experimental steel was input into the formula △σ_y_ = 37 [Mn] + 83 [Si] + 59 [Al] + 38 [Cu] + 3750 [N] + 4570 [C] to calculate solid solution strengthening contribution values at 1000 °C, 1150 °C, 1200 °C reheating temperatures, yielding values of 150, 136, and 116 MPa.

The contribution of precipitation strengthening to yield strength can be determined using the following formula: $△\sigma_{Orowan}=\frac{8.995\times 10^{3}V_{f}^{1/2}}{f}ln(2.417d)$, where V_f_ represents the volume fraction of the precipitated phase, $V_{f}=[\frac{1.4\pi }{6}]\times [\frac{ND_{mean}^{2}}{A}]$. D_mean_ denotes the average diameter of the particles, N is the number of precipitates, and A is the area of the measured image. The V_f_ of Cu-containing precipitates in the test steels at 1000 °C, 1150 °C, 1200 °C reheating temperatures was calculated to be 0.1%, 0.47%, and 1% (Figure 11 and Figure 12). According to the provided formula, the precipitation strengthening contribution values of Cu-containing precipitates in the test steels at 1000 °C, 1150 °C, 1200 °C reheating temperatures were 83, 179, and 215 MPa. The calculated Vf of Nb–Ti carbide precipitates in the test steels at 1000 °C, 1150 °C, 1200 °C reheating temperatures was 0.7%, 0.27%, and 0.21% (Figure 15 and Figure 16). However, the precipitation strengthening effect of the second phase with an equivalent diameter greater than 60 nm was not significant compared with the variations in the proportion of temperatures below 60 nm, reheating temperature of 1000 °C, 1150 °C, 1200 °C test steel ratio of 87%, 60%, 40%. The contribution values of Ti–Nb carbide precipitation strengthening were 35, 17, and 9 MPa for 1000 °C, 1150 °C, 1200 °C reheated experimental steels. Additionally, the precipitation strengthening contribution values for 1000 °C, 1150 °C, 1200 °C reheated experimental steels were 107, 186, and 224 MPa.

**Figure 18:**
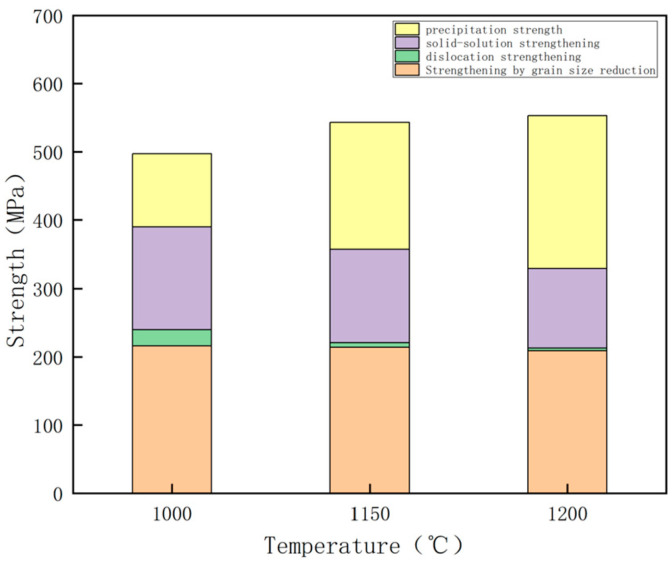
Calculation results of yield strength at different reheating temperatures and the contribution of each part of the strengthening mechanism.

The yield strength of the experimental steel was analyzed by calculating the contribution of each part of the strengthening mechanism, and the results are summarized in Figure 18. The changes in the yield strength of the experimental steel at different reheating temperatures were mainly influenced by precipitation strengthening (Figure 18).

## 4. Conclusions

This study examined the effects of reheating temperature on the mechanical properties and microstructure of 440 MPa grade ship plate steel. The key findings are as follows:

(1) After rolling at 1000 °C, 1150 °C, and 1200 °C, the air-cooled test steel exhibited yield strengths of 468, 568, and 569 MPa, respectively. This indicates an increase in yield strength with higher reheating temperatures. At −40 °C, the impact energy of the steel at 265, 206, and 175 J decreased with increasing reheating temperature. Additionally, the ductile–brittle transition temperature increased with higher reheating temperatures. At a reheating temperature of 1150 °C, the air-cooled test steel after rolling exhibited an optimal balance between strength and toughness. Moreover, the mechanical properties of the steel significantly exceeded the performance index of the new generation 440 MPa grade hull steel.
(2) The microstructure of the test steel mainly comprised ferrite and cementite. With increasing reheating temperature, the grain size of the ferrite matrix increased, leading to an increase in yield strength and a decrease in the impact toughness of the steel. Moreover, with increasing reheating temperature, the size of titanium–niobium carbide precipitates in the original austenite of the test steel increased, while the number of the precipitates decreased. Consequently, this weakened the pinning effect on the grain boundary of the original austenite grain. Moreover, the Cu element gradually dissolved in the original austenite matrix, thereby reducing the hindrance of Cu particles to grain growth. Furthermore, during the subsequent rolling and cooling processes, the size of the original austenite increased, and the ferrite grain size formed through the microstructural transformation also increased.
(3) After rolling, the air-cooled test steel comprised Cu and Ti–Nb carbide precipitated phases. The Cu precipitated phase exhibited a smaller size within the range of 20 nm. Conversely, the titanium–niobium carbide precipitates featured a larger size. With increasing reheating temperature, the number of Cu precipitates in the test steel after rolling and cooling processes increased. Consequently, this resulted in an increased volume fraction, a reduction in the size of the steel, an enhanced precipitation strengthening effect, and a subsequent overall increase in strength. As the precipitated phase in the test steel increased, the elements dissolved in the matrix decreased, leading to a weakening of the solid solution strengthening. Moreover, with increasing reheating temperature, the dislocation density decreased, leading to an increase in the grain size of the ferrite matrix. These changes contributed to a decrease in the impact toughness of the test steel.

## Data Availability

Data are contained within the article.
